# Detection of *Angiostrongylus cantonensis* in the Blood and Peripheral Tissues of Wild Hawaiian Rats (*Rattus rattus*) by a Quantitative PCR (qPCR) Assay

**DOI:** 10.1371/journal.pone.0123064

**Published:** 2015-04-24

**Authors:** Susan I. Jarvi, William C. Pitt, Margaret E. Farias, Laura Shiels, Michael G. Severino, Kathleen M. Howe, Steven H. Jacquier, Aaron B. Shiels, Karis K. Amano, Blaine C. Luiz, Daisy E. Maher, Maureen L. Allison, Zachariah C. Holtquist, Neil T. Scheibelhut

**Affiliations:** 1 Department of Pharmaceutical Sciences, College of Pharmacy, University of Hawaii at Hilo, Hilo, Hawaii, 96720, United States of America; 2 USDA APHIS Wildlife Services, National Wildlife Research Center, Hawaii Field Station, Hilo, Hawaii, 96720, United States of America; 3 Smithsonian Conservation Biology Institute, National Zoological Park, Front Royal, Virginia, 22630, United States of America; Public Health Research Institute at RBHS, UNITED STATES

## Abstract

The nematode *Angiostrongylus cantonensis* is a rat lungworm, a zoonotic pathogen that causes human eosinophilic meningitis and ocular angiostrongyliasis characteristic of rat lungworm (RLW) disease. Definitive diagnosis is made by finding and identifying *A*. *cantonensis* larvae in the cerebral spinal fluid or by using a custom immunological or molecular test. This study was conducted to determine if genomic DNA from *A*. *cantonensis* is detectable by qPCR in the blood or tissues of experimentally infected rats. F1 offspring from wild rats were subjected to experimental infection with RLW larvae isolated from slugs, then blood or tissue samples were collected over multiple time points. Blood samples were collected from 21 rats throughout the course of two trials (15 rats in Trial I, and 6 rats in Trial II). In addition to a control group, each trial had two treatment groups: the rats in the low dose (LD) group were infected by approximately 10 larvae and the rats in the high dose (HD) group were infected with approximately 50 larvae. In Trial I, parasite DNA was detected in cardiac bleed samples from five of five LD rats and five of five HD rats at six weeks post-infection (PI), and three of five LD rats and five of five HD rats from tail tissue. In Trial II, parasite DNA was detected in peripheral blood samples from one of two HD rats at 53 minutes PI, one of two LD rats at 1.5 hours PI, one of two HD rats at 18 hours PI, one of two LD rats at five weeks PI and two of two at six weeks PI, and two of two HD rats at weeks five and six PI. These data demonstrate that parasite DNA can be detected in peripheral blood at various time points throughout RLW infection in rats.

## Introduction

The nematode *Angiostrongylus cantonensis* is a rat lungworm, a zoonotic pathogen that is the cause of human eosinophilic meningitis and ocular angiostrongyliasis. This nematode was first discovered in China in 1935 [1], is endemic in Asia, Australia, the Caribbean islands and the Pacific Islands and has been detected on the American continent and Brazil with more than 2,800 cases of human infection reported in 30 countries [2, 3]. It is the cause of a global, emerging infectious disease commonly known as rat lungworm disease (RLWD). Hawaii Island is the epicenter for RLWD in the United States. According to the Hawaii Department of Health, more than 90% of all statewide cases originate from the rural east side of Hawaii Island.

Rats, primarily *Rattus rattus* and *R*. *norvegicus*, are the definitive host [4]. After ingesting L3 stage larvae, the larvae migrate to the central nervous system of rats where they develop into preadult worms [5]. The worms migrate to the pulmonary arteries, develop to sexual maturity and lay eggs. The eggs hatch into L1 stage larvae in the lung tissues, migrate up the bronchial tree, are swallowed, and 6–8 weeks after infection are excreted with feces [6]. Slugs or snails are the obligatory intermediate hosts for *A*. *cantonensis*. They acquire the L1 stage larvae from consuming rat feces and support parasite development from the first to the third larval stage. L1–L3 stage *A*. *cantonensis* can also be found in the tissues of other kinds of hosts that are passive carriers. In these animals, called paratenic hosts, development of the nematodes does not fully progress [7]. However, third-stage nematodes in paratenic hosts can cause angiostrongyliasis if the L3 are consumed by humans. The most important paratenic hosts are crustaceans (such as prawns and land crabs) and predacious land planarians (such as flatworms in the genus *Platydemus*) (reviewed in [8]). Rats (especially *R*. *rattus* and *R*. *norvegicus*) and gastropods are globally established and especially abundant in urban and agricultural areas. Humans can become infected by ingesting intermediate or paratenic hosts containing infective larvae. Larvae penetrate the intestinal mucosa, then travel via the bloodstream through the liver and lungs to the central nervous system (CNS) [2]. Angiostrongyliasis in humans can range from relatively mild and self-resolving (headache, meningismus, vomiting, nausea and paresthesias) to severe (paralysis, blindness, and fatal encephalitic meningitis) with a characteristic eosinophilia of the peripheral blood and cerebrospinal fluid [7]. Adult humans are generally referred to as “dead-end” hosts, meaning the parasite does not typically reproduce in adult humans but remains in the CNS or can move to the eye chamber (causing ocular angiostrongyliasis), where larvae remain motile in tissues until parasite death [3]. While relatively rare, eosinophilic pneumonitis caused by *A*. *cantonensis* has been reported in adult humans, demonstrating that under some circumstances the parasite is able to leave the CNS and migrate in the bloodstream to the lungs [9, 10]. Reports in pediatric cases also show adult parasites in the cerebral spinal fluid (CSF) of young children and that adult worms can migrate to the lungs causing significant pulmonary complications (G. Erdem MD, Kapiolani Medical Center for Women and Children, Honolulu HI, pers. comm., [9, 11, 12]).

Most human infections are presumed to be due to ingestion of infected hosts (e.g. slugs, snails or other) on contaminated fresh produce. The incubation period ranges from one day to several months [2]. Diagnosis of angiostrongyliasis in humans is generally difficult and involves both clinical evaluation and laboratory testing. The use of therapies such as anthelmintics remains controversial, but reducing inflammation (i.e. via use of steroids) appears critical in infections in adults [13]. A spinal tap (lumbar puncture) is sometimes carried out to relieve pressure on the brain and to look for evidence of infection, but the nematodes are often not detectable in cerebrospinal fluid [13, 14, 15]. Elevated eosinophil count in the blood is another, but less definitive, indication of disease. Computed tomography (CT) scans and magnetic resonance imaging (MRI) methods allow the detection of lesions in the brain, but are generally not useful for differential diagnosis [9, 16, 17]. Clinical symptoms caused by *A*. *cantonensis* can be used to make a presumptive diagnosis, but some symptoms can also be caused by other tissue-migrating helminths, e.g., *Gnathostoma* spp, *Paragonimus* spp and *Taenia solium* metacestodes [18, 19, 20]. Definitive diagnosis can only be made by finding and identifying *A*. *cantonensis* larvae or young adults in the CSF of patients, by using an immunological test to detect RLW antigens or antibodies against RLW antigens, or a molecular test (e.g., quantitative PCR or qPCR) to detect parasite DNA in the CSF, blood, or other tissues. Detection of *A*. *cantonensis* DNA by qPCR has yet to be reported in human blood, but a successful diagnosis was achieved using PCR to detect *A*. *costaricensis* (a related abdominal parasite) DNA in human blood [21] and *A*. *vasorum* in dogs [22]. Here, we report the successful detection of *A*. *cantonensis* in rat blood and peripheral tissues by qPCR at various time points throughout RLW infection.

## Methods

### Animals

A total of 61 wild adult rats (*Rattus rattus*) were captured by live trapping from Waiakea Forest Reserve on the east side of Hawaii Island. Eleven of these were mated to produce the 21 F1 offspring used in this study. Only F1 progeny born in captivity were used to ensure they were not previously infected with *A*. *cantonensis*. F1 progeny were at least four months old before starting the trial to allow for immune system maturity and decline of maternal antibodies which are reported to be undetectable by 6–8 weeks [23]. Because the parents mated at different times, the exact age of the F1 progeny involved in the study differed somewhat ranging from four months, 14 days to five months, 13 days at the start of the study. We optimally matched the treatment groups in terms of ages and weights where possible. Animals were housed in 36 cm x 17.5 cm x 17.5 cm stainless steel wire cages for individuals, and mating pairs in 42 cm x 24 cm x 17.5 cm stainless steel wire cages. Animals were fed commercial rodent chow (Purina 5001) and provided water *ad libitum*.

### Gavage

Two trials were run and each of the two trials had two treatment groups and one control group: the low dose (LD) treatment consisted of rats which were experimentally infected by gavage of ~10 larvae in one ml of a 0.5% HCl and 0.5% pepsin solution. The high dose (HD) treatment rats were gavaged with ~50 larvae in one ml HCl/pepsin solution. The uninfected control (C) group received only the one ml HCl/pepsin solution containing no larvae. Larvae were isolated from semi-slugs (*Parmarion martensi*) on the same day using the Baerman filtration technique [24]. The adult slugs were collected from a farm in the Puna district of Hawaii Island where we have demonstrated a high prevalence of infected slugs. Larvae isolation involved finely chopping the slugs with a razor blade and placing the slug tissue in 25 ml solution of 1% HCl and 1% pepsin for 2–3 hours on a mechanical agitator at 37°C to digest away slug tissue. The solution was then filtered through a Kimwipe in a glass funnel with a collection tube with clamp attached. Larvae in the continuous fluid column migrated down through the filter and accumulated in the tube in a flocculent layer just visible above the clamp. After 1–2 hours, 5 mls of solution was drawn from the collection tube and larvae were counted in gridded petri dishes. Diluted solutions of ~10 larvae/ml and ~50 larvae/ml were then prepared based on manual larval solution density counts. For gavage, rats were anesthetized, manually restrained, and one ml of appropriate solution (LD, HD or Control) was administered by syringe and gavage tube directly into their stomachs.

### Blood Sample Collection

To collect blood samples, rats were anesthetized in their cages in a devised anesthesia chamber using isoflurane-soaked cotton balls in a 50 ml Falcon tube. While anesthetized, rats were placed in commercially available restrainers designed for tail bleeding (Braintree Scientific, Braintree MA). Most blood samples were collected by tail bleeding using heparinized 22 g butterfly needles with the extension tube clipped off so the blood would drip directly into a heparinized collection tube. Cardiac bleeding was conducted post-euthanasia at six weeks PI using a heparanized 21 g needle and syringe. For all blood samples collected, 100 μl of whole blood was placed into DNA lysis buffer (0.1 M Tris-HCl, 0.1 M EDTA, 2% SDS) for molecular testing. All tubes were stored at -80°C. Tail tissue for DNA extraction was collected after euthanizing. All tools used for tail tissue collection were either sterile, single use blades, or thoroughly cleaned and bleached between each use. Tails were excised and then degloved in a biosafety cabinet. Interior tail sections were collected and placed in DNA lysis buffer and frozen at -80°C.

Blood samples were collected from a total of 21 rats throughout the course of two trials; 15 rats were involved in trial I and six rats in trial II. The most comprehensive description of the life cycle of an *Angiostrongylus* species was by Mackarras and Sandars [25]. Therefore the time points for collecting blood samples used Mackerras’ and Sandars’ [25] description of the parasite *(A*. *mackerrasae*) life cycle in the rat as a guideline. The nematode is expected to enter the rat’s bloodstream in the first few hours post-infection (PI) by piercing the intestinal wall and to then remain in the bloodstream for up to 24 hours. Generally within one hour, L3 larvae can enter venules and be transported to the liver, then eventually to the right atrium of the heart and lungs and into the main circulatory system [13]. From the circulatory system, larvae can move directly into the CNS, or other tissues like the kidneys or musculature, but by 4 days PI, no larvae have been found outside the CNS [13]. Many time points were thus selected within the first 24 hours PI as well as during days two and three PI. Once in the CNS larvae mature from L3 to L5 stage. All larvae are expected to be in the CNS and brain by day four PI and can remain there until approximately 26 days PI while they mature, so minimal testing was completed during this time period. When they reach maximum development and size (length ~12 mm) in the brain, they migrate out and enter the circulatory system and travel back to the heart and lungs (approximately 26–33 days PI).

### DNA Extraction

All DNA extractions were performed using the Qiagen DNeasy Animal Blood and Tissue Kit with slight modifications from the manufacturers’ suggested protocol. Whole blood or tail tissue sections in lysis buffer were thawed completely. One-half of the volume of blood (approximately 50 μl each whole blood and DNA lysis buffer) and approximately 100–200 mg of tail tissue in lysis buffer were combined with 40 μl Proteinase K and a variable volume of Buffer ATL for a total digestion volume of 400 μl. Following overnight digestion at 55°C with gentle shaking (~60 rpm), samples were combined with 400 μl Buffer AL and 400 μl 100% ethanol. Extractions then proceeded according to standard protocol, with elution in 200 μl AE repeated once for a total volume of 400 μl gDNA. Each batch of extractions included a single extraction blank (additional Buffer ATL instead of blood) that was carried through qPCR as well.

### Quantitative PCR (qPCR)

Extracted DNA was subjected to qPCR using species-specific primers for the internal transcribed spacer (ITS1) region [26]. The assay used for qPCR was a Custom TaqMan Gene Expression Assay (Life Technologies, Grand Island, NY, assay ID: A139RIC) and carried out as described in Jarvi et al. [27]. Cycling conditions were 94°C for 30 sec, 65°C for 30 sec, and 72°C for 1 min with 45 cycles. Each sample was run at least twice, using two different volumes. In the first reaction, samples were run at 2 μl and 5 μl, using a set of standards with a range of DNA equivalence of 0.071–49.7 *A*. *cantonensis* L3. Following evaluation of the data from the first reaction, all further reactions used template volumes of 5 μl and 9 μl using standards ranging from 0.0071–35.5 *A*. *cantonensis* L3. All samples run in the first reaction were repeated using the second reaction conditions for consistency. All samples producing a signal in any reaction were repeated again.

### Sequencing

To confirm qPCR produced the expected product, PCR products from seven rats were isolated in a 2% low-melt gel, and gel extractions completed using a NucleoSpin kit (Macherey-Nagel) according to manufacturer’s protocols. Products were sequenced at the University of Hawaii at Hilo EPSCOR Core Genetics Facility.

At the end of both trials all rats were euthanized in a CO_2_ chamber. Rats were necropsied, and measurements recorded for body weight, spleen weight and length. Adult nematodes were extracted from the rat heart and lungs, sexed and counted. Statistical analyses for comparisons of interest were conducted by One-way ANOVA in Minitab V. 16 or a 2-sample Wilcoxon test in R.

### Ethics Statement

All procedures in this study were completed in accordance with the University of Hawaii Institutional Animal Care and Use Committee’s (IACUC), which approved this study (protocol #12-1433-2) and USDA-Animal Plant Health Inspection Service-National Wildlife Research Center, which approved this study (QA-1998). USDA/Animal Plant Health Inspection Service/Wildlife Services has a standing permit to trap rats at Waiakea Forest Reserve and on other state lands. The University of Hawaii IACUC adheres to the Guide for the Care and Use of Laboratory Animals in accordance with the Public Health Service requirements, the Animal Welfare Regulations in accordance with the Animal Welfare Act, and to the US Government Principles for the Utilization and Care of Vertebrate Animals Used in Testing, Research, and Training. Animals were anesthetized using isoflurane soaked cotton balls in an anesthesia chamber. At the end of the trials, rats were euthanized in a CO_2_ chamber.

## Results

**Fig 1:**
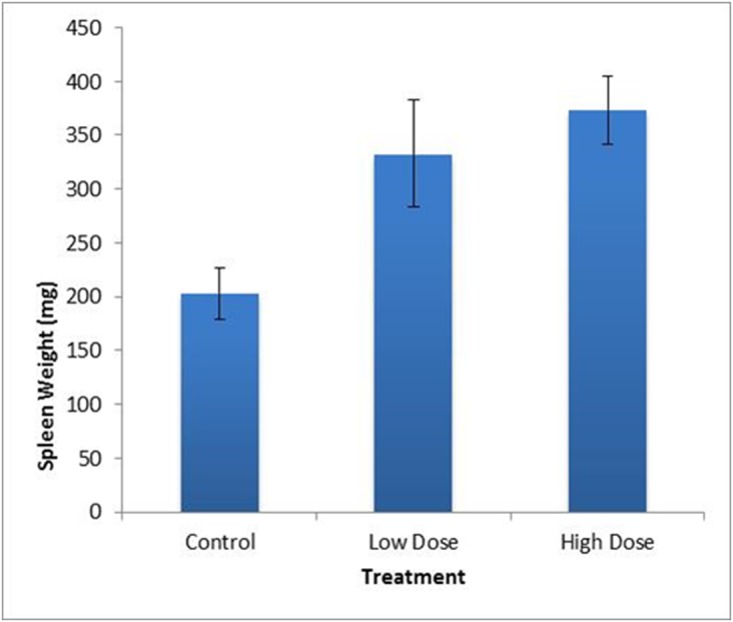
Mean spleen weights (g) of control, low dose and high dose rats. Mean spleen weight of the HD was significantly greater (*p* = 0.011, F = 5.92. DF = 2) than the uninfected controls. Mean spleen weight of control rats was 202.8 mg, of LD rats was 332.9 mg and of HD rats was 373.3 mg. Standard error bars are indicated.

**Fig 2:**
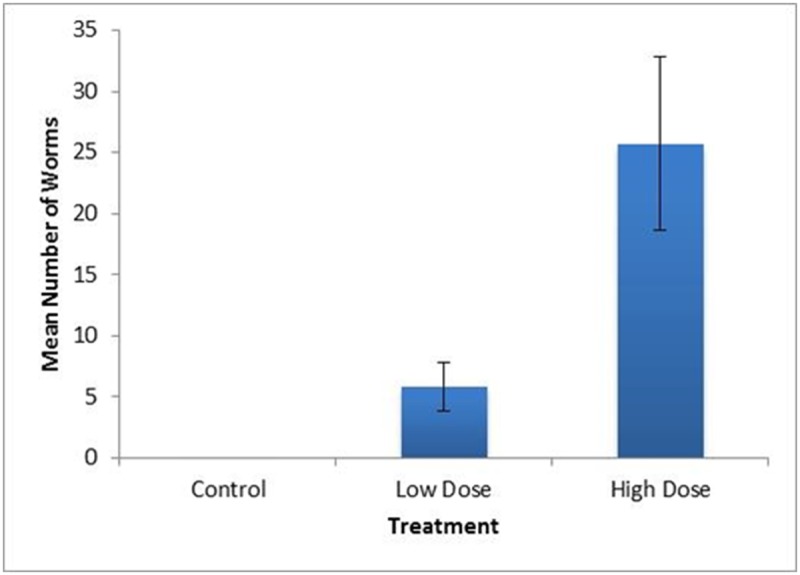
Mean number of adult worms isolated from heart and lungs. The mean number of adult worms isolated from the heart and lungs of HD rats was significantly higher (*p* = 0.001, F = 10.11, DF = 2) than that isolated from LD or control groups. Worm numbers ranged from 0–16 in the LD group and 8–57 in the HD group. Mean worm numbers of control rats was 0, of LD rats was 5.9, and of HD rats was 26. Standard error bars are indicated.

A total of 21 wild rats produced in captivity were involved in the two trials described in this study. Variables measured throughout the trials are summarized in Table 1. We found no significant difference in change in body weight between pre-infection and six weeks PI among the three groups (control, low dose, high dose) (*p* = 0.317, F = 1.22, DF = 2), although the high dose group tended to show the lowest average change in total body weight after infection. Mean spleen weight of the high dose treatment was found to be significantly greater (*p* = 0.011, F = 5.92. DF = 2) than the uninfected controls (Fig 1), however, spleen length did not differ among groups (*p* = 0.262, F = 1.44, DF = 2) (Table 1). The mean number of adult worms isolated from the heart and lungs of high dose rats was significantly higher than those isolated from low or control treatments (*p* = 0.001, F = 10.11, DF = 2) (Fig 2). Worm counts ranged from 0–16 in the low dose group and 8–57 in the high dose group. No worms were isolated from individuals in the control group. The mean number of worms in females was lower in the LD group (4.25, n = 4) than in males (8, n = 3) but not significantly (*p* = 0.59). However the reverse was seen in the HD group where mean number of worms in females was greater (26.8 n = 6) than in the male (19 n = 1), but this observation is undoubtedly influenced by the inadvertent sex skew in the HD group.

**Table 1:** Summary of the rat identification and measurements recorded during the trial including gender, number of worms recovered at necropsy, body weights prior to infection and at 6 weeks PI, spleen measurements and treatment group (C control, LD low dose, HD high dose).

Trial	Rat ID	Gender	Number of worms*	Body wt** (g) pre-infection	Body weight (g) 6 wks PI	Change in body weight	Spleen weight (mg)*	Spleen length (mm)	Treatment
I	42	M	0	156.0	176.3	20.3	162.2	22.0	C
I	46	m	0	169.8	170.2	0.4	144.8	21.7	C
I	71	f	0	163.8	198.3	34.5	277.8	26.8	C
I	75	m	0	150.2	160.7	10.5	210.0	22.7	C
I	80	m	0	144.5	170.8	26.3	196.6	24.2	C
II	165	m	0	157.9	179.6	21.7	133.0	25.0	C
II	157	f	0	137.4	115.1	-22.3	295.0	25.0	C
	Mean				167.3	13.1	202.8	23.9	
I	43	m	4	153.9	153.5	-0.4	193.7	21.0	LD
I	47	f	2	140.0	165.8	25.8	279.9	26.5	LD
I	72	f	6	186.8	197.6	10.8	369.7	28.6	LD
I	76	m	4	179.7	190.3	10.6	421.0	26.5	LD
I	96	f	9	169.9	162.4	-7.5	293.0	24.8	LD
II	127	f	0	150.8	160.2	9.4	208.0	22.0	LD
II	133	m	16	207.0	226.6	19.6	565.0	33.0	LD
	Mean		5.9		179.5	9.8	332.9	26.1	
I	44	f	8	133.6	130.3	-3.3	241.1	24.1	HD
I	48	f	21	133.0	137.2	4.2	451.4	28.7	HD
I	73	f	12	143.5	145.0	1.5	338.2	27.6	HD
I	77	f	57	130.8	128.8	-2.0	350.0	23.6	HD
I	82	f	16	137.4	153.4	16.0	388.6	28.0	HD
II	123	f	47	145.8	143.8	-2.0	500.0	28.0	HD
II	132	m	19	144.7	145.6	0.9	624.0	39.0	HD
	Mean		26		140.6	2.2	413.3	28.4	

*The mean number of adult worms isolated from HD rats was significantly greater (*p* = 0.001, F = 10.11, DF = 2) than those isolated from LD or C groups. The mean spleen weight of HD rats was significantly greater (*p* = 0.011, F = 5.92. DF = 2) than uninfected controls.

** Rats in trial I were ~5 months of age and rats in trial II were ~6 months of age at the time of body weight measurement.

Adult worms were recovered from chambers of the heart in some rats, but the majority of adult worms were recovered from the circulatory vessels inside the lungs. A difference in lung tissue was often observable between rats with zero or low numbers of worms in the lungs and those rats with higher worm counts. Generally, as contrasted with relatively small, light pink, elastic healthy lung tissues, the lungs of heavily infected rats appeared swollen, granular and inelastic with blotchy coloration suggestive of inflammation, consistent with observations by Makarras & Sandars [25]. Areas at the tips of alveolar sacs filled with eggs hatching to L1 larvae and L1 larvae bursting through the tissues into the airspace of the lungs appeared yellowish to tinged and stippled with black.

Positive qPCR results were found in all of the experimentally infected rats in Trial I (both LD and HD treatments) at six weeks PI using DNA extracted from cardiac blood, as well as three of five LD and five of five HD rats from tail tissue samples (Table 2). PCR products originating from tail tissue from three LD and four HD rats were direct sequenced and were 100% identical to each other; confirmation that they represent the ITS1 5.8S ribosomal gene region was done by Blast searches in GenBank. The sequence is reported here instead of in GenBank as they do not meet the minimum length requirement of at least 200 nucleotides for submission. The 60 base pair nucleotide sequence is: GGGTCATTAAGATTTCCTGTCAATCAGGTGTCACATGCGTATAGTAGATATGCGATGATA. In Trial II, parasite DNA was detected in peripheral blood samples from one of two HD rats at 53 min. PI, one of two LD rats at 1.5 hours PI, one of two HD rats at 18 hours PI, one of two LD rats at five weeks PI, two of two LD rat at six weeks PI, and two of two HD rats at weeks five and six PI (Table 2). These results demonstrate that parasite DNA can be detected in peripheral blood or tissue at various time points throughout RLW infection in wild rats.

**Table 2:** Results of qPCR for detection of RLW DNA in blood or tissue of rats involved in Trial I and Trial II.

Trial I n = 5 rats/treatment				Trial II n = 2 rats/treatment		
Treatment	Time PI	Number of qPCR replicates/ rat^1^	Number of rats positive^2^	Time PI	Number of qPCR replicates/ rat^3^	Number of rats positive^2^
Control	pre-bleed	4	0	pre-bleed	4, 8	0
	4–7 hrs	4	0	45–55 mins	4	0
	21–24 hrs	4	0	2–2:20 hrs	4	0
	day 2	4	0	14–15 hrs	4	0
	day 3	4	0	16–17 hrs	4	0
	week 1	4	0	18–19 hrs	4	0
	week 2	4	0	Week 4	2, 4	0
	wk 6 cardiac	2	0	Week 5	2	0
	wk 6 tail	8	0	Week 6	2, 4	0
Low dose	pre-bleed	4	0	pre-bleed	2	0
	4–7 hrs	4	0	30–35 mins	2	0
	21–24 hrs	4	0	1:30 hrs	2, 6	1
	day 2	4	0	14–15 hrs	2	0
	day 3	4	0	16–17 hrs	2	0
	week 1	4	0	18–19 hrs	2	0
	week 2	4	0	Week 4	2, 4	0
	week 6 cardiac	2	5	Week 5	4	1
	wk 6 tail	12	3	Week 6	2, 4	2
High dose	pre-bleed	4	0	pre-bleed	2	0
	4–7 hrs	4	0	50–60 mins	2, 6	1
	21–24 hrs	4	0	2–2:15 hrs	2	0
	day 2	4	0	14–15 hrs	2	0
	day 3	4	0	16–17 hrs	2	0
	week 1	4	0	18–19 hrs	2	1
	week 2	4	0	Week 4	2	0
	week 6 cardiac	2	5	Week 5	4, 5	2
	wk 6 tail	12	5	Week 6	2	2

High dose infection (HD, 50 larvae/ml gavage), low dose (LD, 10 larvae/ml gavage), control (C, 0 larvae/ml gavage).

^1^One extraction per individual except wk 6 tail: tail tissue, which had two extractions per individual for controls, 3 extractions per individual for infected.

^2^Positive = clear signal in large proportion of replicates

^3^If the number of replicate qPCR tests differed between the two individuals, they are reported separately.

## Discussion

In rats, the lungworm larvae or remnants of larvae appear to spend a significant amount of time in the circulatory system, thus RLW DNA should be detectable in the blood at certain stages. Parasite DNA was detected at three time points within the first 18 hours PI in Trial II, and at both weeks 5 and 6 PI in trial II and at six weeks in trial I. Selection of time points for blood sampling was based on the *A*. *mackerrasae* life cycle description of Mackerras and Sandars [25]. The results of this study demonstrate that qPCR can detect parasite DNA in blood samples collected during some time periods predicted to be the windows of sampling opportunity during which the parasite would most likely be circulating in blood [25].

The highest spleen weight was observed in the HD treatment group, as might be expected. The greatest numbers of adult worms were also predictably found in rats in the HD treatment group. The HD group was also unintentionally female biased. Simoes et al. [28] reported that prevalence of *A*. *cantonensis* was higher in female rats as compared to males in the natural population they studied. In our LD group, which had a more equivalent ratio (4 females: 3 males), there was no significant difference in mean number of worms (*p* = 0.59). The small sample sizes and sex bias of the HD group in this study undermine any conclusive interpretation based on sex. We isolated 16 adult worms from the heart and lungs of one LD rat and 57 in one HD rat. The infecting dose was estimated to be either 10 or 50 larvae/ml, respectively, but each dose could vary slightly, as they all were pulled from master solutions estimated to contain approximately either 10 or 50 larvae/ml. The finding of 57 adult worms in the heart and lungs means that at least 57 L3 to L5 stage larvae resided in the CNS and brain for several weeks prior to migrating to the heart. While we observed a reduced (but not significantly reduced) weight gain in the HD group, we observed no other abnormal behaviors suggesting extreme pain or distress in these rats. In this study, ingestion of ~50 larvae in rats is referred to as a high dose, but in the wild we have found slugs infected with nearly 7,000 larvae [29], so rats in the wild are likely infected by much higher doses than 50 larvae. It has been reported that in light or moderate infections, histological evaluation of the brain showed no evidence of cerebral damage at any stage due to live larvae, including movement through the grey matter, however, inflammatory cells were observed surrounding dead larvae and cast skins [25]. Several studies have since shown that killing the larvae may increase inflammation and likely the severity of disease (reviewed in [13]). It is unclear how live larvae can migrate throughout the rat brain with little or no obvious detrimental effects or immune system stimulation, while dead larvae and cast skins result in increased inflammation. One explanation could be an increase in immunogenicity as the larvae die and break down, resulting in increased inflammation. Another explanation might be that live larvae secrete immunomodulatory or other molecules resulting in a decreased inflammatory response or produce molecules that are protective of the live larvae in the brain. These molecules would cease to be produced as larvae die, which could result in increased inflammation.

After the first trial was completed (Trial I), a smaller second trial (Trial II) was conducted to test blood samples collected during time periods not included in Trial I. In Trial I, blood samples were collected twice within the first 24 hour period (between 4–7 hours and 21–24 hours PI) (Table 2). None of these samples were positive for parasite DNA based on qPCR results. Blood samples were also collected on days two and three PI, and during weeks one, two and six PI. In Trial II, blood samples were collected five times within the first 24 hour period PI (two bleeds within the first 3 hours PI, and three bleeds between 14–19 hours PI), and during weeks four, five and six PI (Table 2). Parasite DNA was detected early at 53 minutes PI and 1.5 hours PI. This finding demonstrates how quickly the parasite traverses the mucosal membrane and enters the bloodstream. It is unknown if the prior exposure to HCl/pepsin during isolation influenced this result. Parasite DNA was not detected between 1.5 hours PI and 17 hours PI, but was detected at 18–19 hours PI. This suggests variability in the time it takes for parasites to traverse the mucosa. There is likely variability in the time it takes for parasites to traverse the mucosal membrane and enter the bloodstream in human infections as well.

In recent studies, researchers have been able to isolate parasite DNA in the blood of canines infected with *A*. *vasorum*, the cause of canine angiostrongylosis, using a combined approach of a real time PCR coupled with an ELISA test [22]. PCR was recently used for clinical diagnosis of *A*. *cantonensis* infection from cerebral spinal fluid [30]. Additionally, a successful diagnosis was achieved using a PCR method to detect *A*. *costaricensis* (a related South American abdominal parasite) DNA in human blood (21). The accidental ingestion of an infected intermediate host allows *A*. *cantonensis* L3 stage larvae to infect humans by penetrating through their intestinal mucosa and traveling via the bloodstream through the liver and lungs until the parasite reaches the CNS (2). There is likely variability in how long the parasite takes to cross the intestinal membrane and travel to the brain in humans via the bloodstream. Parasite DNA might be detected during this transit. Additionally, some parasites may become lodged in the intestinal wall and become stationary due to mucosal immunity. Under immune attack, these parasites might begin to shed cells releasing DNA into the bloodstream for an indeterminate amount of time. Within the first 24 hours PI, L3 larvae have been found in the mesenteric lymph gland of a rat [25]. Also, as was found in this study and others [25], there is clear discrepancy between the number of larvae given and the number of adult worms later isolated from the heart and lungs. This suggests that many larvae can be ‘lost’ during the initial migration. The larvae that do not make it to the brain would likely succumb to immune attack, thus releasing additional DNA into the bloodstream over time. Evidence exists that parasites can leave the human CNS and migrate via the bloodstream to the lungs (9, 10, 11,12, G. Erdem MD, Kapiolani Medical Center for Women and Children, Honolulu HI, pers. comm.). These observations suggest other possible time points for detection of parasite DNA in the blood by qPCR. This study demonstrates that parasite DNA can be detected in peripheral blood at various time points throughout infection in rats which could be useful in studies aimed at quantifying the RLW contamination rate of rats in an area. Additionally, this technique may be useful as a confirmatory diagnostic in humans and could allow for early detection in cases of known accidental raw gastropod or paratenic host ingestion.
